# Supplementary material for: The Management of Children with Cancer during the COVID-19 Pandemic: A Rapid Review
Source: J Clin Med. 2020 Nov 21;9(11):3756. doi: 10.3390/jcm9113756 (PMC7700610; doi:10.3390/jcm9113756)
Supplement: Supplementary file 1 [file jcm-09-03756-s001.pdf]

Figure S1 - Flow chart of the literature review

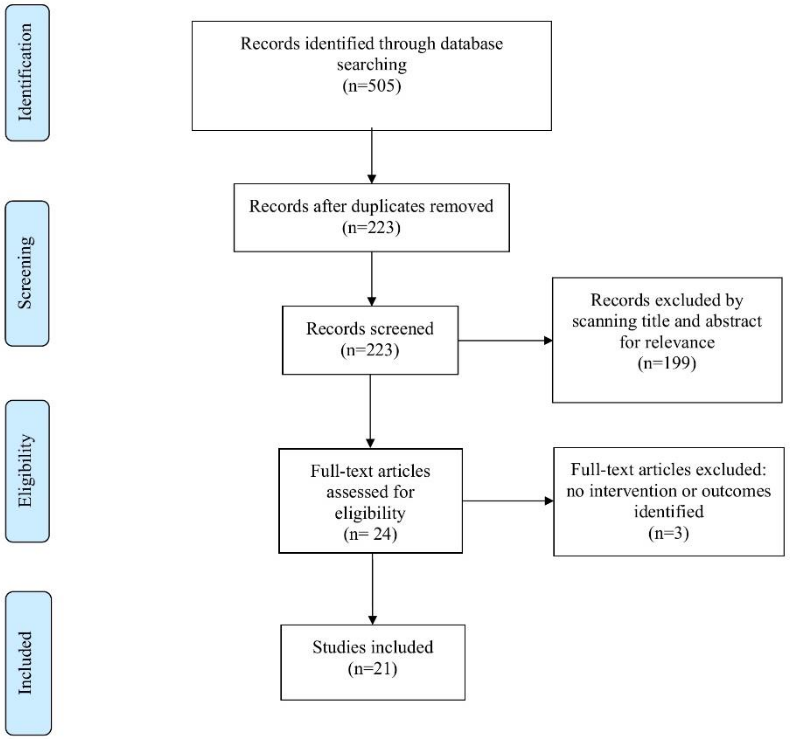

Figure S1. Flow chart of the literature review.

Supplementary Table S1 - Search Strategy

Database	Search strategy	Identified Articles
PUBMED	#1 (((((((“Child"[MeSH Terms]) OR "Children*") OR "Adolescent"[MeSH Terms]) OR "Pediatric?") OR "Pediatrics"[MeSH Terms]))) <br>#2 (((((((“cancer”) OR “tumor”) OR “neoplasms” [MeSH Terms]) OR "Medical Oncology” [MeSH Terms]) OR “Hematology” [MeSH Terms]) OR “Oncology Service, Hospital” [MeSH Terms], OR “Oncology”)) <br>#3 #1 AND #2 <br>#4 (((((((((((((((“Education” OR “prevention[MeSH Subheading] AND control”[MeSH Subheading])) OR "Intervention*") OR "prevention*”) OR “Secondary Prevention”[MeSH Terms]) OR “Management*”) OR “Disease Management”[MeSH Terms]) OR (“Organization and Administration”[MeSH Terms])) OR “Health Information Management”[MeSH Terms]) OR “Risk Management”[MeSH Terms]) OR “restraint*”) OR (“Organization and Administration"[MeSH Terms])) OR “Management Service Organizations”[MeSH Terms]) OR “Organization*”) OR "clinical strategies*") OR "managing*")	188

	#5 ((((((("COVID-19") OR "SARS-CoV-2") OR "nCoV") OR "2019 novel coronavirus") OR "2019-nCoV") OR "novel coronavirus"))<br><br>#6 #3 AND #4 AND #5	
SCOPUS	S1 ("Child*" OR "Adolescent" OR "Pediatric?" OR "Pediatrics")<br><br>S2 ("cancer*" OR "tumor*" OR "neoplasms*" OR "Medical Oncology" OR "Hematology?" OR "Oncology Service, Hospital" OR "Oncology")<br><br>S3 S1 AND S2<br><br>S4 "education" OR "Intervention*" OR "prevention*" OR "Management*" OR "Disease Management" OR "Health Information Management" OR "Risk Management" OR "restraint*" OR "Management Service Organizations" OR "Organization*" OR "clinical strategies*" OR "managing*"<br><br>S5 "COVID-19" OR "SARS-CoV-2" OR "nCoV" OR "2019 novel coronavirus" OR "2019-nCoV" OR "novel coronavirus"<br><br>S6 S3 AND S5 AND S5	144

COCHRANE LIBRARY	Search by topic and by *review group*	3
EMBASE	(("Child*" OR "Adolescent" OR "Pediatric?" OR "Pediatrics") AND ("cancer*" OR "tumor*" OR "neoplasms*" OR "Medical Oncology" OR "Hematology?" OR "Oncology Service, Hospital")) AND (“Education” OR "Intervention*" OR "prevention*" OR "Management*" OR "Disease Management" OR "Health Information Management" OR "Risk Management" OR "restraint*" OR "Management Service Organizations" OR "Organization*" OR "clinical strategies*" OR "managing*") AND ("COVID-19" OR "SARS-CoV-2" OR "nCoV" OR "2019 novel coronavirus" OR "2019-nCoV" OR "novel coronavirus")	170

Supplementary Table S2 - COVID-19 interventions by description in the included studies.

	Preventive interventions														
Author	Screening	PPE	Hand hygiene	Isolation	Management: Chemotherapy (a), Radiotherapy (b), Surgery (c)	Telephone support (d), Online support (e), Telemedicine (f)	Limitation: Parents (g), Carers (h), Volunteers (i)	Health education	Temperature detection (j), Swabs (k)	Dedicated staff	Waste disposal	Psychological support	Pulmonary CT (l), Nucleic acid test (m)	Dedicated pathways (n), Move follow-up visits (o) Visit bedside (p)	HSCT postponement
Subspecialty Group of Hematology and Oncology, 2020	X	X	X	X	a	e,f	g	X	j,k			X	l		
Yang et al., 2020	X	X	X	X	a,b,c			X	k		X				
Kotecha, 2020	X	X	X	X		g	g,h,i		k					o	
Bouffet et al., 2020	X	X	X	X		d,e,f,	g		k					o	
Balduzzi et al., 2020	X	X	X	X	a	d,e	g,i	X	j,k	X		X		n,o,p	X
He et al., 2020	X	X	X	X	a,b,c		h	X	k	X	X		m	n	
Hrusak et al., 2020	X	X	X	X	a,b				j,k	X					
Saab et al., 2020		X	X	X	a,b,c									n,o	
Cassoux, 2020			X	X										n,o	
Manjandavida et al., 2020					a,b,c									n,o	
Trehan et al., 2020				X		d,e				X				n,o	

Cai et al., 2020	X	X	X	X	a,c			X		X				n	
Iehara et al., 2020	X		X	X	a,b										X
Sullivan et al., 2020		X	X	X	c	d,f	g,h	X	k	X	X	X		o	X
Blinded, Yearb	X	X	X	X		d	g,h,i							n,o	X
Baruchel et al., 2020		X	X	X	a,b,c				k				l,m		
Ruggiero et al., 2020		X	X	X	a	f	g,h			X					
Seth, 2020		X	X	X	a,b,c					X				n,o	
Blinded, Yeara		X	X	X				X	k		X				
Kaspers, 2020				X			g,h		k					o	
Sainati & Biffi, 2020	X	X	X	X	a	d	g,h,i	X	j,k	X	X			n,o	X

Abbreviations:

PPE, personal protection equipment

CT, computed tomography

HSCT, Hematopoietic stem cell transplant
